# EMBER creates a unified space for independent breast cancer transcriptomic datasets enabling precision oncology

**DOI:** 10.1038/s41523-024-00665-z

**Published:** 2024-07-09

**Authors:** Carlos Ronchi, Syed Haider, Cathrin Brisken

**Affiliations:** 1 ISREC - Swiss Institute for Experimental Cancer Research, School of Life Sciences, Ecole Polytechnique Fédérale de Lausanne (EPFL), CH-1015 Lausanne, Switzerland; 2 The Breast Cancer Now Toby Robins Breast Cancer Research Centre, The Institute of Cancer Research, London, UK

**Keywords:** Cancer genomics, Breast cancer, Predictive markers

## Abstract

Transcriptomics has revolutionized biomedical research and refined breast cancer subtyping and diagnostics. However, wider use in clinical practice is hampered for a number of reasons including the application of transcriptomic signatures as single sample predictors. Here, we present an embedding approach called EMBER that creates a unified space of 11,000 breast cancer transcriptomes and predicts phenotypes of transcriptomic profiles on a single sample basis. EMBER accurately captures the five molecular subtypes. Key biological pathways, such as estrogen receptor signaling, cell proliferation, DNA repair, and epithelial-mesenchymal transition determine sample position in the space. We validate EMBER in four independent patient cohorts and show with samples from the window trial, POETIC, that it captures clinical responses to endocrine therapy and identifies increased androgen receptor signaling and decreased TGFβ signaling as potential mechanisms underlying intrinsic therapy resistance. Of direct clinical importance, we show that the EMBER-based estrogen receptor (ER) signaling score is superior to the immunohistochemistry (IHC) based ER index used in current clinical practice to select patients for endocrine therapy. As such, EMBER provides a calibration and reference tool that paves the way for using RNA-seq as a standard diagnostic and predictive tool for ER+ breast cancer.

## Introduction

Breast cancer (BC) is the most frequently diagnosed cancer worldwide^1^. More than 70% of all cases are estrogen receptor positive (ER + )^2^, i.e., at least 1% of the tumor cells express ER as assessed by immunohistochemistry (IHC)^3^. According to ASCO guidelines, patients with ER+ BCs receive endocrine therapy. Overexpression of HER2 characterizes about 30% of all patients and is a criterion for anti-Her2 targeted therapies, whilst the Ki67 index is used to determine which patients need chemotherapy in addition to endocrine therapy.

High throughput transcriptomic profiling has refined subtyping of breast cancer^4,5^ and resulted in a number of clinically approved RNA-based molecular signatures, such as Breast Cancer Index, MammaPrint, EndoPredict, Oncotype DX and Prosigna^6–10^. These can help identify ER + BC patients who will benefit from chemotherapy and avoid overtreating those who will not. These signatures, however, provide limited biological insights into why a particular patient needs additional therapy^11^ and they do not assess the benefit of endocrine therapy.

With global transcriptomics becoming increasingly affordable, sequencing platforms, such as the SCAN-B consortium and the Hartwig Medical Foundation already provide mutational and gene expression based biomarkers within one week of tumor surgery for clinical decision making^12–15^. Furthermore, the SCAN-B consortium demonstrated that RNA-seq can be used to determine ER status, intrinsic molecular subtypes^16^, identify targetable mutations^12^, and to calculate risk scores using single sample predictor algorithms within the South Swedish cohort^16^. Hence, it is conceivable that RNA-sequencing may replace more expensive predictive testing and provide more comprehensive information.

Here, we develop a novel and robust data-integration approach allowing us to integrate both microarray and RNA sequencing-based datasets into a common space, molecular EMBeddER, henceforth called EMBER. Cohorts and individual patient samples can be added to the space. The localization of a patient sample in the EMBER space provides a novel way to interpret the molecular subtypes as a continuum and to find candidate biological pathways of resistance to endocrine therapy. Furthermore, we provide evidence that functional ER signaling performs better at predicting response to endocrine therapy than the current clinical standard ER IHC index.

## Results

### EMBER: a statistical framework to integrate different transcriptomic data sets into a common space

**Fig. 1:**
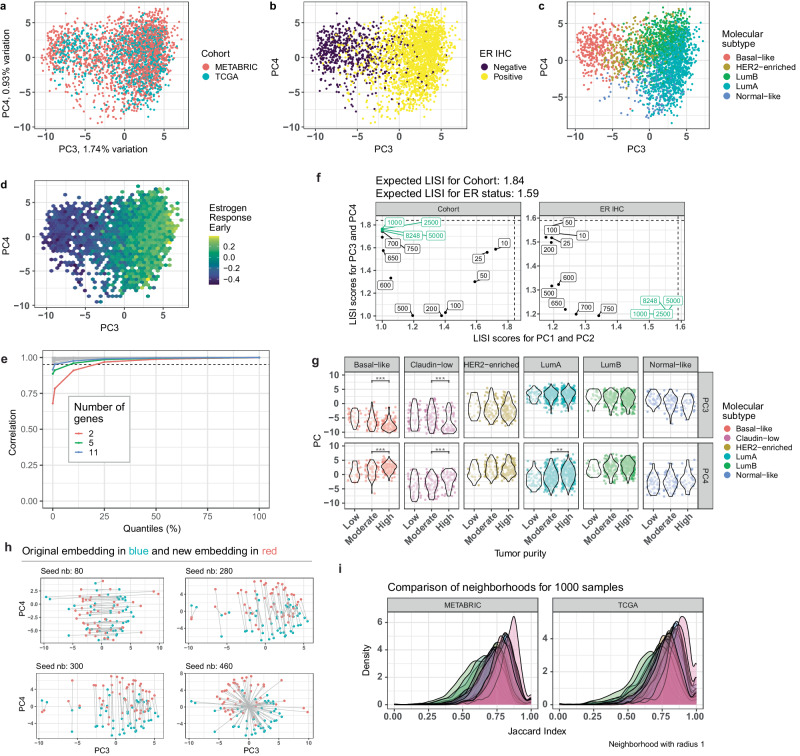
Development of EMBER. **a** EMBER score plot of the principal components (PCs) PC3 and PC4 for TCGA and METABRIC samples. All samples included. Colored by cohort. **b** Same as (**a**) colored by ER IHC. **c** Colored by intrinsic molecular subtype. **d** Hexagon (hex) grid calculated on PC3 and PC4. Each hex is colored based on its average value of the estrogen response early. **e** Quantiles of the correlation when using different number of genes for the stable gene set. Only the gene sets that have minimum correlation below 0.95 are highlighted. **f** Localized Inverse Simpson Index (LISI) scores when considering different sets of genes, ranging from 10 to 8248 with different increments. The green color corresponds to the number of genes that have similar LISI scores in PC1/PC2 and PC3/PC4. The left plot corresponds to the comparison of the LISI scores calculated based on the PC1/PC2 and PC3/PC4 embeddings when using cohort as a feature and the plot on the right when using ER IHC as a feature. **g** Distribution of PC3 and PC4 stratified by molecular subtype and tumor purity using all samples from METABRIC. **h** Embedding of random samples given different training sets for PCA. Blue dots correspond to the original embedding of a sample and red dots correspond to the new embedding given the new training set. **i** Jaccard index distribution for one thousand random samples in each cohort. Each color corresponds to a different seed when sampling for the training. A total of 25 seeds were selected. (ANOVA followed by Tukey’s HDS. *p-value < 0.05, **p-value < 0.01 and ***p-value < 0.001).

To enable the integration of different large transcriptomic datasets, we developed a statistical model (Supplementary Figure 1a) named molecular EMBedER (EMBER). We focused on patients with early stage BC and used both bulk RNA-seq and microarray data from TCGA and METABRIC cohorts, respectively^5,17^. To bring the data from the two different platforms on the same scale, we first selected the 1000 most variable genes in each dataset by the average coefficient of variation, and then divided the rankings by the average ranking of 44 stable genes^18^. The average ranking distribution of the 44 pre-selected stable genes showed slight differences between METABRIC and TCGA. The mean value of the distribution from TCGA is higher than the mean distribution observed in the microarray-based METABRIC dataset (Supplementary Figure 1b), suggesting platform-related differences. Yet, the average ranking of stable genes is the same between ER+ and ER- BC samples across METABRIC and TCGA (Supplementary Figure 1c). Importantly, the normalization process preserved the gene expression levels characteristic for ER+ and ER- BC samples, as exemplified by the expression levels of *ESR1* itself, known to be correlated with ER IHC^19^ and of the ER target gene *TFF1* (Supplementary Figure 1d) found among the 1044 selected genes. We performed a training-test split of the data, using 1000 samples (352 TCGA and 648 METABRIC) for the training and the remaining for testing. To explore the relationship between TCGA and METABRIC samples, score plots were generated using the first four components. The first two principal components (PCs) reflect batch effects related to study cohort and platforms (RNA-seq and microarray) used (Supplementary Figure 1e). In the PC3 and PC4 score plot, samples from both cohorts overlapped indicating tumor intrinsic similarities (Fig. 1a). More specifically, PC3 largely separated tumors by their IHC-based ER status (Fig. 1b). The luminal A and B subtypes were on the right, the basal-like subtype on the left most part of the third component and the HER2-enriched subtype in between the three (Fig. 1c). To further characterize PC3, we color coded EMBER using the GSVA scores obtained from the Hallmark Estrogen Response Early gene set^20^. High values correspond to activated ER signaling and low values correspond to either low or no ER signaling. Samples in the rightmost region of EMBER had higher values compared to those on the left region (Fig. 1d). This gradient highlighted the ER signaling transcriptional differences between ER+ and ER- tumors when using EMBER.

To systematically evaluate the effectiveness of the minimum number of stable genes, we changed the number of stable genes by randomly sampling 2 to 42 genes from the list of 44 stable genes and calculated the normalized gene expression for every gene (1000 genes) on TCGA and correlated (Spearman) it to the original qPCR-like normalized expression. We show that when using 2 genes only, the minimum correlation is 0.68 but the 10% quantile is 0.91. By using more than 11 genes the minimum correlation becomes 0.97 (Fig. 1e). Next, we systematically evaluated the mixing of the two cohorts as a function of the number of genes considered using the Localized Inverse Simpson Index (LISI)^21^. This analysis shows that 1000 genes provide good mixing in PC3/PC4 exclusively (Fig. 1f, Supplementary Figure 2). When fewer genes are used, the mixing deteriorates, while increasing the number of genes beyond 1000 does not improve performance.

As clinical factors, such as tumor stage, nodal status, age, NPI and tumor purity may affect the embedding, we calculated the linear relationship (Pearson ${r}^{2}$) between the clinical factors with PC3 and PC4 (Supplementary Figure 3a–g). Tumor purity from METABRIC did not show strong correlation with PC3 and PC4 when stratified by the intrinsic molecular subtypes (Fig. 1g). The proportion of variance explained by the linear correlation between tumor purity and PC3/PC4 is ${r}^{2}$ = 0.01 (1%) and ${r}^{2}$ = 0.08 (8%), respectively (Pearson ${r}^{2}$, Supplementary Figure 3g), whereas for ER IHC the ${r}^{2}$ with PC3 was 0.5 (50%). Age, inferred menopausal state, Nottingham Prognostic Index (NPI) and node stage accounted for 6%, 5%, 15% and 1% of variance explained in PC3 respectively. Together, all of them accounted for less than 2% in PC4. The NPI is usually correlated to more aggressive BCs, that tend to be of the basal-like subtype^22^, hence it had a higher Pearson ${r}^{2}$ compared to other clinical factors. Taken together, tumor purity and various clinical factors had limited contribution to the embedding.

To understand the effect of centering and scaling the data prior to applying PCA, we compared the loadings of PC1 from EMBER to the average of the genes in the normalized data (Supplementary Figure 4a). The correlation between these two variables is 1, showing that PC1 corresponds to centering. When comparing the loadings with PC2 and the standard deviation of genes after centering, the higher loadings in absolute value are correlated with a higher standard deviation (Supplementary Figure 4b). Next, we show that PC1 and PC2 are correlated with PC1 obtained after centering and scaling of the data (Supplementary Figure 4c), showing that EMBER’s PC1 and PC2 are automatically centering and scaling the data while removing batch effects. In order to confirm that no biological information is lost when performing PCA on the uncentered and unscaled data, we compared EMBER’s PC3 and PC4 with PC2 and PC3 from the scaled data, respectively (Supplementary Figure 4d, e). The correlation is 1 in both cases for both cohorts: METABRIC and TCGA. Thus, there is no need to center and scale prior to applying PCA in EMBER’s context.

Next, we compared EMBER to a conventional batch-effect removal algorithm and an embedding technique: Combat and Autoencoders. We applied Combat (Supplementary Figure 4f) on the 1000 samples from TCGA and METABRIC (logFPKM and median intensity gene expression respectively). PC1 and PC2 from Combat are correlated (−0.99 and −0.98) with EMBER’s PC3 and PC4, demonstrating that EMBER works as well as Combat in removing batch effects. In distinction from Combat, EMBER allows one to add single samples to the embedding and to calculate single sample scores (Fig. 5). When using an autoencoder with a single hidden layer with 2 units on the same samples as Combat, there is no mixing of the cohorts (Supplementary Figure 4g). When expanding to 4 units instead of 2 and using the qPCR-like normalized data, some mixing is observed (Supplementary Figure 4h), although ER+ and ER- BC samples are mixed. We tried different autoencoder architectures (Supplementary Table1), with all of them showing the same mean squared error (0.05).

To assess the robustness of the approach, the analysis was repeated 25 times with different random subsets of 1000 patient samples each from TCGA and METABRIC cohorts. Each iteration of the embedding process either shifted all samples in the same direction or reflected them along a common axis (Fig. 1h). This indicates that the samples used for training do not affect the biological properties captured by EMBER. When systematically testing sensitivity to missing genes, we observed no effect for up to 30% of missing top genes based on the loadings of the third and fourth components (Supplementary Figure 1f, g). To quantitatively evaluate the stability of random subsets, we compared the number of samples that coincide within each neighborhood of random samples to the corresponding neighborhood in the original embedding. The Jaccard index, a measure of how similar two sets are, was on average 0.74 (Standard Deviation: 0.06), indicating a high level of agreement between the neighborhoods (Fig. 1i). In conclusion, different BC transcriptomic datasets can be embedded in a common space, the proposed method is robust and stable, and captures the key features of breast cancer such as ER status and intrinsic molecular subtypes.

### Validation of embedding principles in independent cohorts

**Fig. 2:**
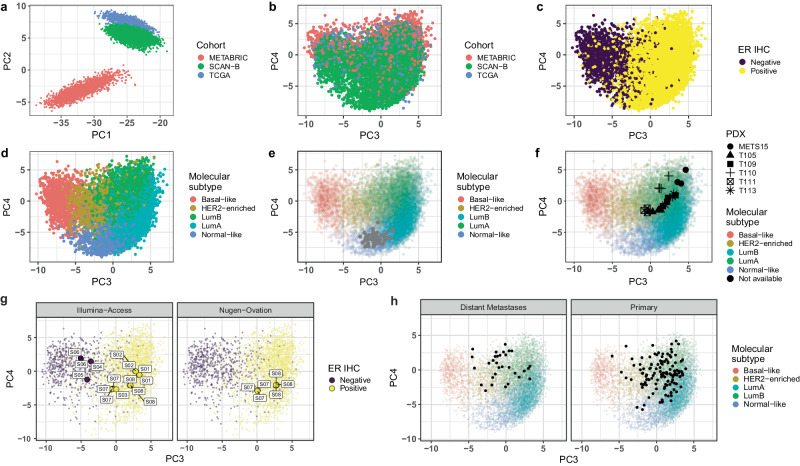
External cohort validation of EMBER. **a** PC1/PC2 score plot using the first and second component of TCGA, METABRIC and SCAN-B. **b** PC3/PC4 score plot of all samples from the three cohorts: TCGA, METABRIC and SCAN-B. **c** Same as **b**, colored by ER IHC. **d** Same as **b**, Colored by intrinsic molecular subtype. **e** PC3/PC4 score plot including samples (in grey) from reduction mammoplasties overlayed on TCGA, SCAN-B and METABRIC colored by the intrinsic molecular subtype. **f** Embedding of six different PDXs (METS15 *n* = 3; T113 *n* = 3; T109 *n* = 4; T105 *n* = 5; T111 *n* = 3; T110 *n* = 4), T113 and T111 being responders, METS15 and T105 partial responders and T109 and T110 non-responders^25^. **g** Embedding of FFPE samples sequenced using different library preparations: Illumina-Access and Nugen-Ovation. The text labels correspond to the sample IDs. **h** Embedding of biopsy samples from the Metastatic Breast Cancer Project dataset either from distant metastases (left) or from the primary site (right).

To validate EMBER, we used the 1044 genes identified in the training sets to integrate a third large breast cancer cohort, SCAN-B^15,16^ into the EMBER space. In the PC1/PC2 score plot (Fig. 2a) SCAN-B samples were closer to the TCGA than to the METABRIC samples, in line with the former two cohorts being RNA-seq based while the latter is microarray-based. In the PC3/PC4 score plot SCAN-B samples overlayed METABRIC and TCGA samples (Fig. 2b), the five intrinsic molecular subtypes were distinguishable (Fig. 2c) and PC3 separated ER+ and ER- BC samples (Fig. 2d), confirming EMBER’s ability to capture molecular subtypes in this independent cohort. Of note, SCAN-B samples were prepared with 3 different library protocols: dUTP, NeoPrep, and TruSeq. Although we used the logFPKM measurements without any library protocol adjustment^23^, EMBER mixed all the samples (Supplementary Figure 3h).

To address the bias towards Caucasian patients in the three cohorts, we next tested EMBER on a South Korean BC cohort^24^ comprising 168 Asian patients with primary BC (Supplementary Figure 5a–c). The samples projected to expected regions according to their molecular subtypes, showing that EMBER can be applied to diverse populations.

Next, we embedded 66 RNA-seq samples from women who underwent reduction mammoplasty in Switzerland^16^. As expected, these samples predominantly fell within the region of the normal-like molecular subtype (Fig. 2e). Finally, we projected RNA-seq samples from a series of intraductal (MIND) ER+ patient derived xenografts (PDXs)^25^ in the EMBER space. Consistent with their selection based on their ability to expand efficiently in mice, they localized to the luminal B region (Fig. 2f).

We extended the analysis to another cohort, including only FFPE samples that were sequenced using different library preparations, Illumina-Access and Nugen-Ovation^26^. Three ER- samples (S04, 05 and 06) locate to the ER- region and three ER+ samples (S01, 02 and 03) to the ER+ region (Fig. 2g), matching their ER IHC status. Two samples, S07 and S08, sequenced using both methodologies embedded to the same location, regardless of library preparation (Fig. 2g). We also applied EMBER to the Metastatic Breast Cancer Project dataset^27^, which consists of matched primary tumor and metastases samples preserved in FFPE blocks. The embedding of all samples stratified by distant or primary site show that they localize mostly in the luminal B and HER2-enriched regions (Fig. 2h). ER+ and ER- BC samples are clustered together with all the ER+ and ER- BC samples from SCAN-B respectively (Supplementary Figure 5d). Thus, EMBER captures the molecular subtypes and can be applied to different BC patient cohorts, MIND-PDXs, FFPE samples as well as metastatic lesions.

### EMBER provides additional biological information for molecular subtyping

**Fig. 3:**
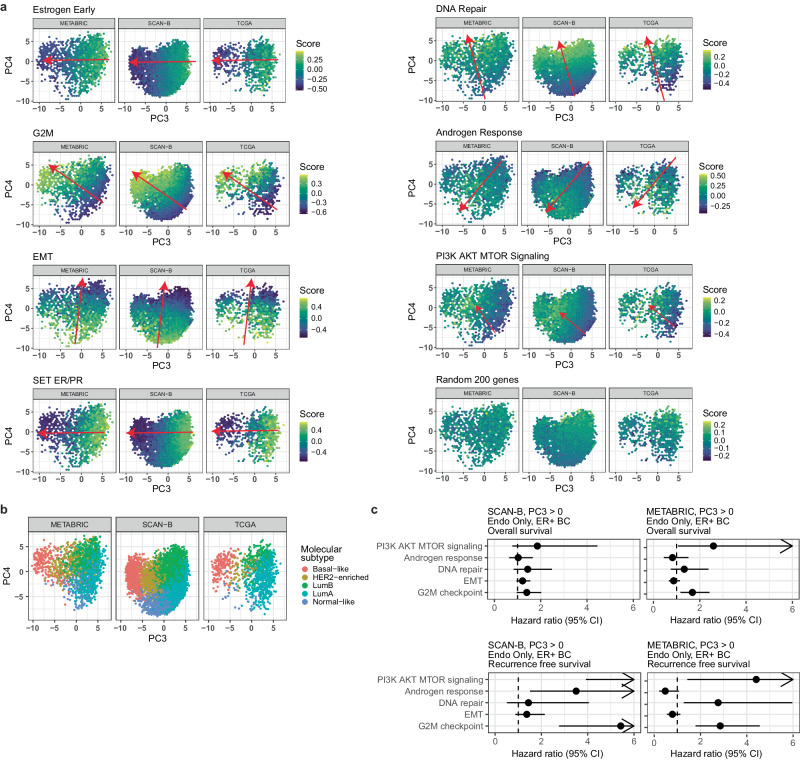
Molecular drivers of EMBER. Average scores per hexagon and survival analysis in three large breast cancer cohorts. **a** PC3/PC4 score plots of all samples from TCGA, SCAN-B and METABRIC grouped in hex regions. Color codes the average GSVA score. Selected pathways are shown. The arrows correspond to the direction of the gradient. **b** PC3/PC4 score plot of TCGA, SCAN-B and METABRIC colored by molecular subtype. **c** Survival analysis results obtained for each pathway. Pathways were adjusted as described in the methods section.

Since the EMBER-derived space captures a continuous spectrum of heterogeneity in patient samples well beyond the 5 molecular subtypes of BC, we hypothesized that the precise location of a sample may provide additional biological information. To test this hypothesis, we estimated the activity of molecular pathways, some of which are targeted with existing drugs, using GSVA scores for each cohort, TCGA, SCAN-B and METABRIC separately (Fig. 3a). SET ER/PR and Estrogen Early were represented with high GSVA scores on the rightmost region of the EMBER space and lowest scores in the leftmost part of the EMBER space. Androgen response had higher scores in the HER2 enriched region closer to the normal-like molecular subtype. The gene set with 200 random genes had no specific directionality in the embedding. As expected, G2M checkpoint was higher in the more proliferative luminal B subgroup than in the luminal A subgroup while EMT was higher in luminal A than B. Additionally, DNA repair and PI3K AKT MTOR signaling scores showed gradients across the embedding space with luminal A samples having the lowest scores in both cases (Fig. 3a). Overall, the pathway scores exhibited different directional changes across the two principal components, indicating that they captured information beyond the molecular subtypes (Fig. 3b) in addition to estrogen receptor signaling, cell proliferation pathways and HER2 signaling.

To further test the clinical relevance of sample position in EMBER and the molecular pathway scores, we evaluated them for association with overall and recurrence-free survival for ER + BC patients who received only endocrine therapy (METABRIC: OS/RFS and SCAN-B: OS/RFS). In doing so, we included only luminal A and B molecular subtypes since basal-like tumors are usually ER- and we are focusing on tumors potentially associated with estrogen signaling (Fig. 3c). Survival analysis revealed an association of G2M checkpoint with poor outcome (2.85 RFS METABRIC HR, (1.79-4.56) CI; 5.05 RFS SCAN-B HR, (2.72-9.38) CI; 1.68 OS METABRIC HR, (1.17-2.42) CI; 1.72 OS SCAN-B HR, (1.22-2.41) CI; Fig. 3c; Supplementary Table 2). This is in line with a higher proliferation index being associated with worse outcomes as further reflected in the differences between luminal A and B subtypes^28^. High PI3K/AKT/MTOR signaling pathway activity was also associated with a worse outcome in both METABRIC and SCAN-B cohorts (4.41 RFS METABRIC HR, (1.43, 13.58) CI; 14.97 RFS SCAN-B HR, (3.41-65.61 CI)). The fact that additional pathways drove the embedding, in particular PI3K/AKT/MTOR signaling and DNA repair, and were prognostic in ER + BC suggest that EMBER provides additional biological information.

### Evaluating EMBER in a window trial

To further test EMBER in a real world clinical setting, we used gene expression data from the POETIC trial^29^, a window trial, which evaluated tumor response to the aromatase inhibitors letrozole or anastrozole in postmenopausal ER+ BC patients using Ki67 index as endpoint. For each patient, a core biopsy at baseline and a core or excision biopsy after 14 days of treatment had been collected at surgery. In parallel to global gene expression analysis, Ki67 indices were determined by IHC on adjacent sections. Responders were defined as showing more than 60% reduction in Ki67 index within 2 weeks of treatment.

**Fig. 4:**
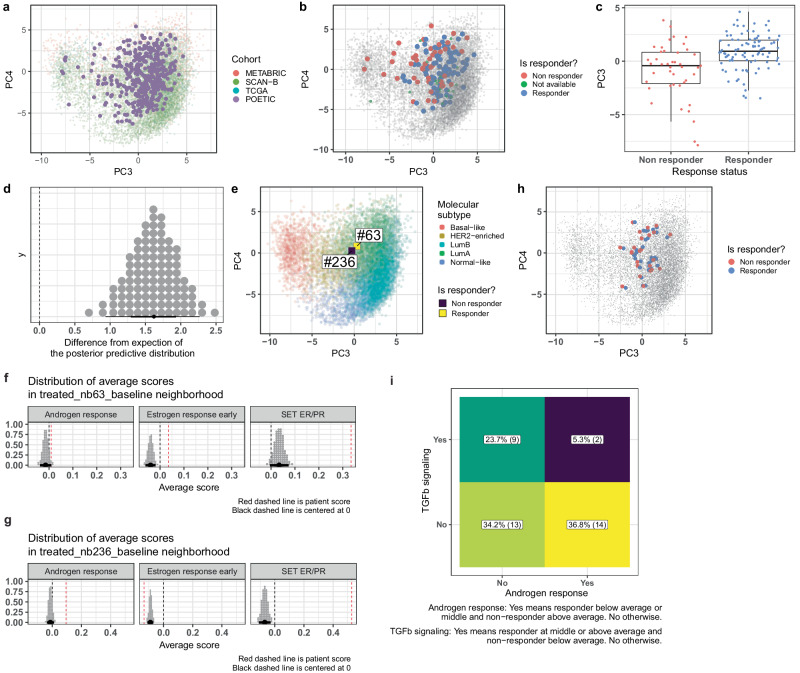
Clinical validation of EMBER. **a** PC3/PC4 EMBER of the POETIC samples at baseline and at surgery colored by cohort. **b** Score plot of the POETIC samples colored by response status with all samples from TCGA, METABRIC and SCAN-B in grey. **c** Comparison of PC3 between all responders and non-responder in the POETIC dataset. **d** PC3 difference in the expectation of the posterior predictive distributions of the responders and non-responders. Each dot corresponds to a percentile. **e** PC3/PC4 Score plot highlighting two patients with similar embedding and different response status. **f** Posterior distributions of the average scores in the neighborhood of the responder patient. The red line corresponds to the patient score. **g** Posterior distributions of the average scores in the neighborhood of the **non**-responder patient. The red line corresponds to the patient score. Systematic comparison for all pairs of responders and non-responders that are close to each other (distance in EMBER ≤ 0.5). For each pathway the sample score was compared to the average posterior distribution in the neighborhood, and it was classified as above the distribution, in the middle of the distribution or below. **h** Embedding of all the 38 pairs of samples that are close to each other. **i** Comparison of Androgen response and TGFβ signaling scores to the average as described previously. The “yes” label for TGFβ signaling represents all the pairs of samples whose non-responder score is below average and the responder score is at the middle or above the average. The “yes” label for Androgen response represents all the pairs of samples whose non-responder score is above average and the responder score is at the middle or below the average.

We hypothesized that the position in EMBER can predict endocrine therapy response and embedded the POETIC trial samples with the TCGA, METABRIC and SCAN-B cohorts (Fig. 4a). As expected, the POETIC samples were closer to the METABRIC samples in the PC1/PC2 score plot (Supplementary Figure 6) because of the common use of microarray platforms. All samples had been classified as ER+BCs by IHC and were hence expected to fall into luminal neighborhood. Yet, they spread across the whole embedding, i.e., also in the basal-like and HER2 enriched regions. Interestingly, the patient samples located outside luminal sample cloud were all non-responders (Fig. 4b). PC3 was predictive of endocrine response (Fig. 4c) and the probability that the PC3 differed between responders and non-responders was 100% with an average difference of 1.5 (Fig. 4d) in line with PC3 being mainly driven by ER signaling.

Yet, within the luminal space multiple non-responders were found (Fig. 4b), we hypothesized that the EMBER space may provide information about factors which determine different endocrine responses in tumors that are close to each other in the embedding. We randomly selected a responder (#63) and non-responder (#236) patient that were in proximity within EMBER (Euclidean distance < 0.5, Fig. 4e) and examined their molecular differences using contextual information by establishing the deviation of their pathway scores from the average scores in their neighborhood (Fig. 4f). The distributions obtained by using Bayesian linear regression corresponded to the average posterior distribution of the scores in the neighborhood, i.e., samples that are in a circle of radius 1 with the respective responder sample as the center. The average score distribution for hallmark estrogen response early in the neighborhood of the selected responder (Fig. 4f) can be interpreted as a 100% probability that the average score is greater than -0.1 and lower than 0. Notably, the responder exhibited a higher estrogen response early score compared to the average in its neighborhood. Conversely, the non-responder displayed a lower estrogen response early score compared to its neighbors, and a higher androgen response signaling score (Fig. 4g). A potential explanation for the differences between the SET ER/PR and Estrogen Response Early scores is that they are not perfectly correlated, 0.48 for SCAN-B and 0.63 for METABRIC in ER + HER2- BC samples. This suggests that differences between two patient samples that are close to each other in the embedding may account for different treatment outcomes.

Next, we analyzed all 38 pairs of samples with a Euclidean distance (PC3 and PC4) of less than 0.5 (Fig. 4h). In total there were 56 samples, 32 responders and 24 non-responders. Notably, these pairs of samples were in the central region of EMBER, i.e., the luminal B and HER2 enriched regions. In 8 of the 38 neighborhoods, the responders had a hallmark estrogen response early score higher than the average while the corresponding non-responder had a score lower than the average. When analyzing the ${SE}{T}_{{ER}/{PR}}$ signature, a measure of ER signaling activity, this number increases to 14. These observations suggest that patients with higher ${SE}{T}_{{ER}/{PR}}$ score than their neighbors are more likely to be responders. We extended this analysis to other pathways, namely androgen signaling represented by Hallmark Androgen Response and TGFβ signaling. In 23.7% (9) of the pairs of responders and non-responders, Hallmark TGFβ signaling was below average for non-responders and at middle or above average for responders. On the other hand, 36.8% (14) of the pairs, Hallmark Androgen Response was higher than average for non-responders and at middle or below average for responders (Fig. 4i). In only 5.3% (2) pairs, the Hallmark TGFβ signaling score was below average and Hallmark Androgen Responder score was above average for non-responder. As such, EMBER provides additional biological information that can be interpreted clinically.

### An absolute scale to compare scores among samples from different cohorts: ASIS

Up to this point, we have compared molecular scores across different large datasets. This was possible because these datasets had similar distributions of clinical factors and intrinsic molecular subtypes. If there is a need to calculate the score from a single sample or a small group of samples from an independently collected dataset as happens both in research and in clinical practice, one is not able to do so using current techniques. A common approach is to use single sample scorers instead, such as singscore and ssGSEA^30,31^. These provide a score that is available on an absolute scale, usually between -1 and 1, but ultimately captures cohort-related effects (Supplementary Figure 7a, b). Since PC3/PC4 from EMBER capture biological effects while PC1/PC2 represent batch effects, we hypothesized we could remove these batch effects and use the batch-free data for downstream analysis.

**Fig. 5:**
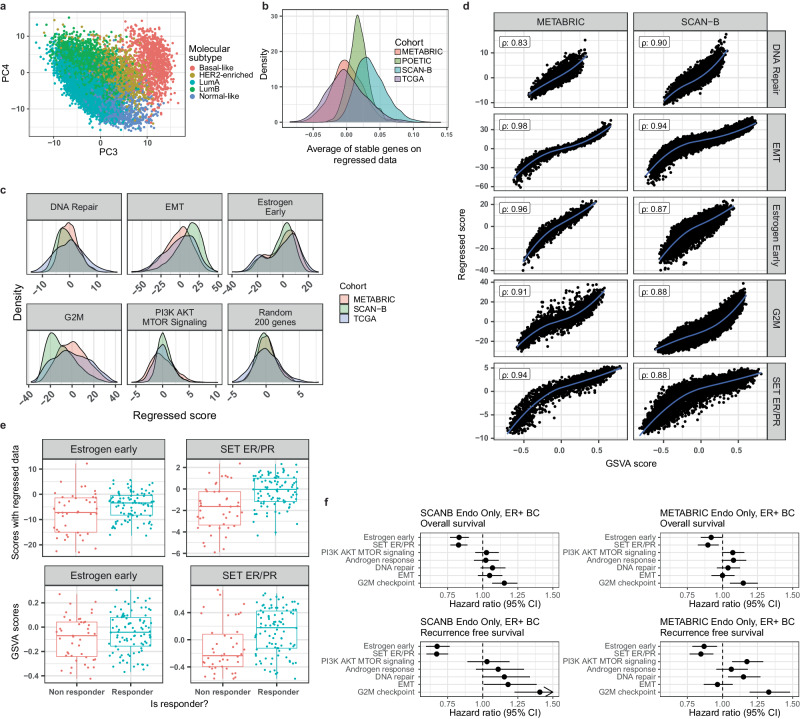
ASIS: An absolute scale for molecular scores. **a** Embedding of all samples when using all 8248 genes shared between TCGA and METABRIC. **b** Average normalized expression of stable genes after regressing out PC1 and PC2. **c** Distribution of pathway scores calculated using all samples from METABRIC, SCAN-B and TCGA. **d** Comparison of GSVA scores on the original dataset with scores obtained using Absolute Scorer for Individual Samples (ASIS). Rho values correspond to the Spearman’s rank correlation between both signatures in each dataset individually. **e** Comparison of the scores obtained from the regressed data (top) and original data using GSVA (bottom) in the POETIC trial dataset. **f** Results of the survival analysis when using the scores obtained from the regressed data.

We recalculated the embedding using the 8248 genes shared between TCGA and METABRIC datasets after filtering instead of the top 1000 most variable genes used previously. The resulting embedding again distinguished the different intrinsic molecular subtypes in PC3 and PC4 (Fig. 5a). To make individual sample scores comparable across different cohorts in a single sample manner, we needed to provide an absolute score scale and thus we defined a new scoring strategy: Absolute Scorer for Individual Samples (ASIS). For this, for each individual sample we regressed the newly created PC1 and PC2 from the data, and then summed the regressed new normalized expression levels of genes in selected gene sets. The sum of the 44 stable genes had consistent values across different cohorts, close to the expected value of 0 (Fig. 5b). Moreover, the distributions of the different molecular pathways fully overlapped when using ASIS (Fig. 5c), improving over the other single sample scorers (Supplementary Figure 7a, b). We next compared the scores for selected hallmark pathways using ASIS, which summed the corrected expression levels of genes, with the original GSVA scores calculated previously for METABRIC and SCAN-B. The signatures derived from both approaches were highly correlated to the original GSVA scores (correlations ranging from 0.83 to 0.98) and had similar ranges across different cohorts (Fig. 5d).

For further biological validation of ASIS, we tested ER signaling scores, Hallmark Estrogen Response Early and ${SE}{T}_{{ER}/{PR}}$ on the POETIC trial data. The estrogen early scores of responders did not overlap with the lower first quartile of the non-responders, in contrast to the GSVA scores that did (Fig. 5e). To evaluate if the scores obtained using ASIS have prognostic power, we performed both OS and RFS analysis on METABRIC and SCAN-B datasets. ER signaling, and proliferation (G2M Checkpoint) signatures, respectively, had hazard ratios below 1 and above 1 (OS and RFS p-values for Estrogen Response Early in SCAN-B: 0.0000066, 5.5e-10; Same for METABRIC: 0.048, 0.0063. For G2M Checkpoint in SCAN-B: 0.00035, 0.0000011; In METABRIC: 0.0017, 2.4e-7), similar to the results obtained when using the GSVA scores, see Fig. 2c (Fig. 5f). Thus, ASIS retains the prognostic power of the molecular signatures and allows for the creation of new signatures that can be easily integrated into the presented framework.

### ER IHC index versus functional ER signaling

**Fig. 6:**
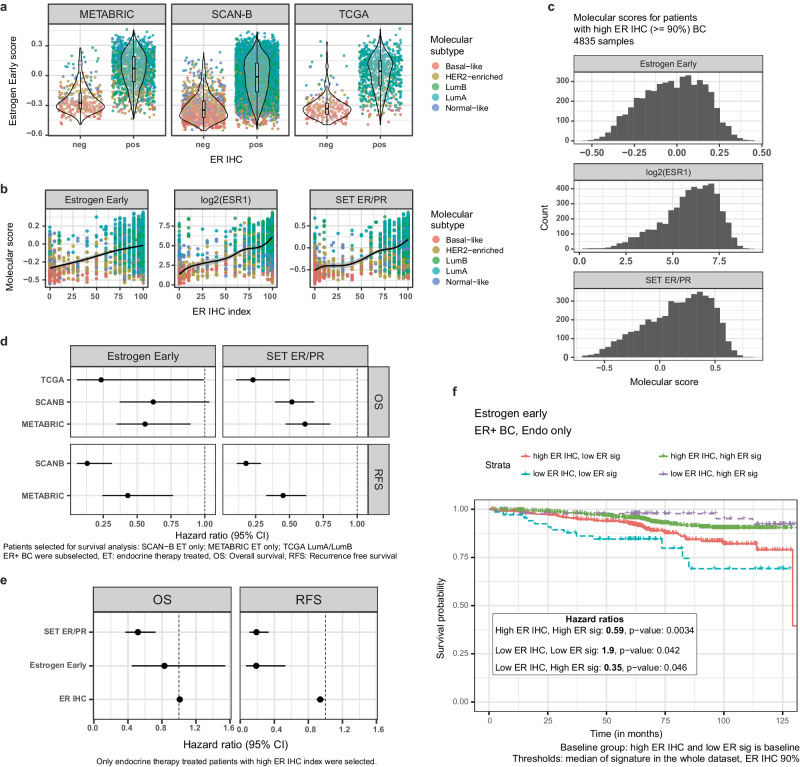
Functional ER signaling vs ER IHC index. Scores and survival analysis results from TCGA, SCAN-B and METABRIC cohorts. **a** GSVA scores by ER IHC for the Estrogen early signature in each patient cohort. Dots represent patient samples. **b** Correlation of ER IHC index with estrogen related molecular signatures on the SCAN-B cohort. **c** Distribution of the estrogen related molecular scores, Hallmark Estrogen Response Early, log_2_(ESR1) and ${SE}{T}_{{ER}/{PR}}$ among patients with high ER IHC index ( ≥ 90%) from SCAN-B. **d** Forest plot of the survival analysis for each cohort separately. **e** Forest plot of the survival analysis for high ER IHC index BC patients that were treated only with endocrine therapy from SCAN-B. **f** RFS analysis on SCAN-B patients treated with only endocrine therapy. ER IHC index was dichotomized at 90% and Estrogen early with the median in all samples of the dataset. NPI: Nottingham prognostic index. Ti: i-th stage of tumor. Ni: i lymph nodes with breast cancer cells.

Our observation that EMBER predicts failure to respond to endocrine in samples that scored ER+ by IHC suggested that ER signaling activity as transcriptional readout may better predict response to endocrine therapy than the current clinical standard, the ER IHC index. We determined ER scores for bulk RNA-seq samples from tumors of patients who received only endocrine therapy using the estrogen signatures HALLMARK_ESTROGEN_RESPONSE_EARLY and HALLMARK_ESTROGEN_RESPONSE_LATE from the molecular signature database^20^ (MSigDB), containing around 200 genes each, and *SET*_*ER/PR*_^32^, which has 18 genes associated with estrogen and progesterone receptor (PR) signaling. Across signatures, more than 75% of genes in the gene sets were available for calculating the scores. The individual scores for patient samples ranged from -0.6 to 0.4 in the three datasets, TCGA, SCAN-B and METABRIC^5,16,17^ (Fig. 6a). The scores ranged from -0.6 to 0 in the ER- and from -0.6 to 0.4, in the ER+ subgroup, with 40 to 50% of all samples from ER+ tumors having a score lower than 0 in each cohort separately.

Metadata from the SCAN-B cohort contains the ER IHC index allowing us to calculate the correlation between this clinically used index and the ER signaling scores. Spearman correlations with ER IHC index were 0.39, 0.51 and 0.57 for Estrogen Early, ${SE}{T}_{{ER}/{PR}}$ and *ESR1* gene expression, respectively (Fig. 6b). Surprisingly, among patients with ER IHC index higher or equal to 90%, the distribution of ER signaling scores corresponded to the overall distribution (Fig. 6c) suggesting a possible mismatch between ER protein expression and functional readout of ER signaling.

Due to the possible mismatch between ER IHC index and functional ER signaling, we hypothesized that the molecular signatures may also be prognostic and provide additional prognostic information for patients with high ER IHC index. To test this, we analyzed ER + BC patients treated with endocrine therapy alone to exclude any effect of chemotherapy or targeted therapy, such as trastuzumab, for SCAN-B and METABRIC. For TCGA, we used luminal A and B samples only without treatment stratification. Some patients in the TCGA cohort might have received chemotherapy, possibly biasing the results available for TCGA. In all the three cohorts, the Hallmark Estrogen Response Early signature was associated with favorable outcome (Fig. 6d; OS TCGA: 0.23, 0.05–0.99 CI; OS SCAN-B: 0.62, 0.37–1.03 CI; RFS SCAN-B: 0.13, 0.05–0.31; OS METABRIC: 0.56, 0.34–0.89 CI; RFS METABRIC: 0.43, 0.24–0.76). Similarly, ${SE}{T}_{{ER}/{PR}}$, hazard ratios also associated with favorable outcome (Fig. 6d; OS TCGA: 0.23, 0.11–0.50 CI; OS SCAN-B: 0.52, 0.39–0.68 CI; RFS SCAN-B: 0.18, 0.11–0.29; OS METABRIC: 0.61, 0.47–0.80 CI; RFS METABRIC: 0.45, 0.33–0.62). When excluding all HER2+ patients, even though they did not receive any targeted therapy for HER2, ER signaling is still prognostic of overall survival and recurrence free survival (Supplementary Figure 8). Thus, functional ER signaling signatures are prognostic in these cohorts both in OS and RFS. Next, we selected only patients with high ER IHC index ( ≥ 90%) from the SCAN-B cohort and re-performed survival analysis (Fig. 6e). The HR for ER IHC index was 1 (OS, CI 0.98–1.04). Both estrogen signatures were associated with a good outcome, with HALLMARK_ESTROGEN_RESPONSE_EARLY OS: 0.83, 0.45–1.54; RFS: 0.18, 0.06–0.52 and ${SE}{T}_{{ER}/{PR}}$ OS: 0.52, 0.37–0.72; RFS: 0.19, 0.11–0.33. This suggests that some patients with high ER IHC index have low functional ER signaling and are likely less responsive to endocrine therapy. Conversely, patients with ER signaling low/ER IHC low BC had a worse outcome than patients whose tumors had high ER signaling and ER IHC low BC (Fig. 6f). Thus, the transcriptomic-based ER signatures were both prognostic and predictive and provided additional information beyond current clinical standard ER IHC index.

## Discussion

Integrating molecular data from various sources presents a significant challenge and is a hurdle in advancing precision medicine. Previous attempts at data integration across distinct technical platforms were restricted solely to cell lines^33^ and did not encompass single-sample-based approaches^34^. Here, we have overcome these problems in the early breast cancer setting with the data integration method EMBER using large public patient datasets such as TCGA, METABRIC, SCAN-B and other cohorts. Although the samples were from different patient populations, sequenced on different platforms at different times, the embedding method captures molecular subtypes and additional biological features. Since the list of stable genes and the normalization procedure remain agnostic to cancer type, our method holds the potential to be applied to different cancer patient cohorts, creating diverse single-sample cancer-specific molecular embeddings. Moreover, different normalization strategies could also be further investigated for possible potential improvements in the embedding.

Most single sample molecular subtype predictors were developed using microarray data, however, when comparing their classifications in the same datasets, they show modest agreement at best, with the exception being the basal-like subtype^35^. This heterogeneity in classifier performance greatly hampers clinical application, where reproducibility is essential. By not assigning a singular molecular subtype to a sample but rather a position in the EMBER space, we embrace the characteristics of a sample on a continuous scale thereby providing a high-resolution view of the entire neighborhood.

Interestingly, the EMBER mapping depends on several known biological pathways, such as estrogen signaling and cell proliferation. Epithelial-mesenchymal transition (EMT) strongly influences the fourth component of the embedding, meaning that luminal A and normal-like tumor samples are in a more EMT state compared to the more proliferative luminal B samples. An intriguing possibility to be explored is that this points to differential plasticity^36^ underlying the biological differences between luminal A and B tumors. Further clinically relevant pathways such as G2M checkpoint, E2F targets and PI3K/AKT/MTOR signaling drive the position on EMBER; drugs are available which target these pathways but are currently only approved in the metastatic setting. Our data suggest that such combination treatments may benefit some early-stage BC patients. In line with this, the prespecified interim analysis of the phase III NATALEE trial (NCT03701334) shows the benefit of a CDK4/6 inhibitor (ribociclib) in combination with ET compared to ET alone for early stage ER+ BC patients at risk for recurrence^37^. For ER+ advanced breast cancer patients, the randomized clinical trial CAPItello-291 showed the added benefit of the Akt inhibitor Capivasertib in combination with Fulvestrant^38^, presenting a possible treatment regime for early stage ER+ BC patients.

Matching EMBER analysis of genomics data with clinical information from AI treated patients in the POETIC window trial^39^ provided additional insights into pathways determining the response to endocrine therapy. We observed that high androgen receptor signaling or low TGFβ signaling scores were associated with a poor response to endocrine therapy. The androgen receptor is tightly linked to the ER in breast cancer, the two receptors compete for DNA binding sites, and their signaling pathways cross talk at multiple levels^40^. More specifically, high androgen receptor expression was found in tamoxifen-resistant tumors^41^.

The definition of ER positivity is a matter of discussion; while ASCO recommends a 1%^3^ threshold, in some countries a threshold of 10% is used^42^. Patients with ER-low BC, i.e. IHC index between 1 and 10% were found to benefit less from endocrine therapy^43^ compared to patients with tumors with an ER index of 10% or more. By demonstrating that functional ER signaling constitutes a continuous measure and that the entire spectrum of signaling scores is observed in tumors with a high ER IHC index ( ≥ 90%), we provide a new functional interpretation of ER signaling applicable to all breast tumors. The results align with published data showing that even among patients bearing tumors with a high ER IHC index, a proportion experience limited response to treatment, classifying them as poor responders^43^.

Further decreases in sequencing costs may make the RNA-seq approach acceptable in the future. With the development of new therapies, more refined and powerful tools will be required for clinical decision making. This is challenging with molecular markers for diagnosis that are difficult to compare across different cohorts in a unified and open manner^35^. The SCAN-B project^15,16^ has shown that it is feasible to have a pipeline for RNA-sequencing in a clinical context. We have shown here how all the data generated in large independent cohorts can be combined and used both in clinical practice and research, further enabling the use of genomics. Our tumor agnostic pipeline may assist the use of RNA-seq in other areas of oncology and precision medicine.

## Methods

### Cohorts

The publicly available breast cancer cohorts TCGA, SCAN-B, and METABRIC^5,15,17^ were used to calculate the principal component analysis (PCA) embeddings and for validation. POETIC^39^ was used as a clinical application of the embedding. From TCGA, SCAN-B and METABRIC, only primary breast cancer samples were included. Each cohort has 1005, 7216 and 1868 primary samples respectively, totaling 10089 samples. The POETIC trial comprises post-menopausal women with primary breast cancer estrogen receptor positive status by IHC, with a total of 426 samples. TCGA is an RNA-seq based cohort, SCAN-B is also RNA-seq based with three different type of library protocols (dUTP, NeoPrep and TruSeq). METABRIC and POETIC are microarray based (Illumina HumanHT-12 v3 and v4 BeadChips respectively). The FFPE dataset contains 6 FFPE samples, from which 3 are estrogen receptor positive (ER + ) BC and 3 are ER- BC samples. The Metastatic Breast Cancer Project is a patient driven project with RNA-seq data of 153 FFPE samples in total, including primary, local, and distant metastasis.

TCGA counts data were downloaded from Firebrowse. SCAN-B data version 3 (StringTie FPKM Gene Data unadjusted) were downloaded from Mendeley https://data.mendeley.com/datasets/yzxtxn4nmd^23^ and one sample was selected for each patient. For patients with multiple samples, the corresponding sample was selected using the following rules: tumor is from the primary site, it has the most aligned pairs, and it has the highest NanoDrop concentration (ng/ul). METABRIC median intensity and z-normalized data were downloaded from cBioPortal. POETIC data and FFPE samples were downloaded from GEO (accession code GSE105777 and GSE130397 respectively) using the R package GEOquery^44^. For POETIC the normalized intensity was used for downstream analysis. For the FFPE samples, logCPM was calculated from the RSEM counts using the function *cpm* from the package edgeR^45^ (v3.40.2). For the Metastatic Breast Cancer Project, RSEM data were downloaded from cBioPortal along with the clinical information and logCPM was calculated using the function *cpm* from the package edgeR^45^. TCGA data was processed following the steps available in the singscore AML mutations workflow analysis^30^ and all downstream analysis were performed on the TMM logFPKM values. The median intensity was used for METABRIC and logFPKM for SCAN-B. For details about downloading and preprocessing steps see https://chronchi.github.io/transcriptomics.

### Selection of highly variable genes and normalization and PCA embedding

The coefficient of variation (CV) was calculated for each gene and in each cohort separately:

1 $${\rm{CV}}=\frac{{\rm{standard\; deviation}}}{{\rm{average\; expression\; level}}}$$

The 1000 genes with the highest average CV in the TCGA and METABRIC cohorts were selected. In addition, 44 genes with stable expression across different cancers^18^ were added. All 1044 genes were ranked from lowest to highest expression for each sample, rankings were then divided by the average ranking of the 44 stable genes in analogy to qPCR normalization to bring all samples to a similar scale. Subsequently, PCA was performed on a total of 1000 random samples (fixed seed) from TCGA and METABRIC, using PCAtools^46^ in R without centering and scaling. Additional individual samples were embedded by multiplying the PCA loading matrix with the sample normalized expression. Samples with less than 1044 genes available were normalized by assigning missing genes a value of 0. During training and testing of EMBER no genes were missing.

### Scoring strategies

For TCGA, SCAN-B, METABRIC and POETIC, Gene Set Variation Analysis (GSVA)^47^ was applied along with the ${SE}{T}_{{ER}/{PR}}$ signature^32^ and the Hallmark collection from the molecular signature database^20,48^. Default parameters were used in the gsva function from the GSVA package^47^. For scoring the cohorts, the logFPKM was used for TCGA and SCAN-B, median intensity for METABRIC and normalized intensity for POETIC. GSVA was used on datasets with genes filtered by expression in each cohort separately.

### Average neighborhood scores

In order to calculate the posterior distribution of the average scores in each neighborhood, a linear regression with only intercept (score ~ 1) was fitted using rstanarm^49^ and the function stan_glm for each pathway individually. When applying the function stan_glm, we used four chains and a prior normal distribution with location 0 and scale equals to 1. The package tidybayes^50^ was used to extract the draws in a tidy format.

### Survival analysis

Cox regression with the survival package from R was used. Variables used for adjustment were age, tumor stage, and number of positive lymph nodes in the case of TCGA and SCAN-B, age, and the Nottingham Prognostic Index (NPI) in the case of METABRIC. Overall survival analysis was performed for all three cohorts and recurrence free survival analysis for METABRIC and SCAN-B.

### R package

EMBER is available as an R package at https://github.com/chronchi/ember with a tutorial including the pathway scores with the regressed data, installation instructions and further documentation.

### Supplementary information

Supplemental Material

## Data Availability

All the data used in this study are already publicly available and referenced.

## Code availability

The code used to generate all the analysis is available at https://github.com/chronchi/molecular_landscape. Instructions on how to fully reproduce the figures and analysis in this paper, run the docker image are available at the previously mentioned repository on GitHub. An online version with a website containing all the analysis and code can be found at https://chronchi.github.io/molecular_landscape.
