# Supplementary material for: EMBER creates a unified space for independent breast cancer transcriptomic datasets enabling precision oncology
Source: NPJ Breast Cancer. 2024 Jul 9;10:56. doi: 10.1038/s41523-024-00665-z (PMC11233672; doi:10.1038/s41523-024-00665-z)
Supplement: Supplementary file 1 — Supplemental Material [file 41523_2024_665_MOESM1_ESM.pdf]

Supplementary Data

Supplementary Figure 1

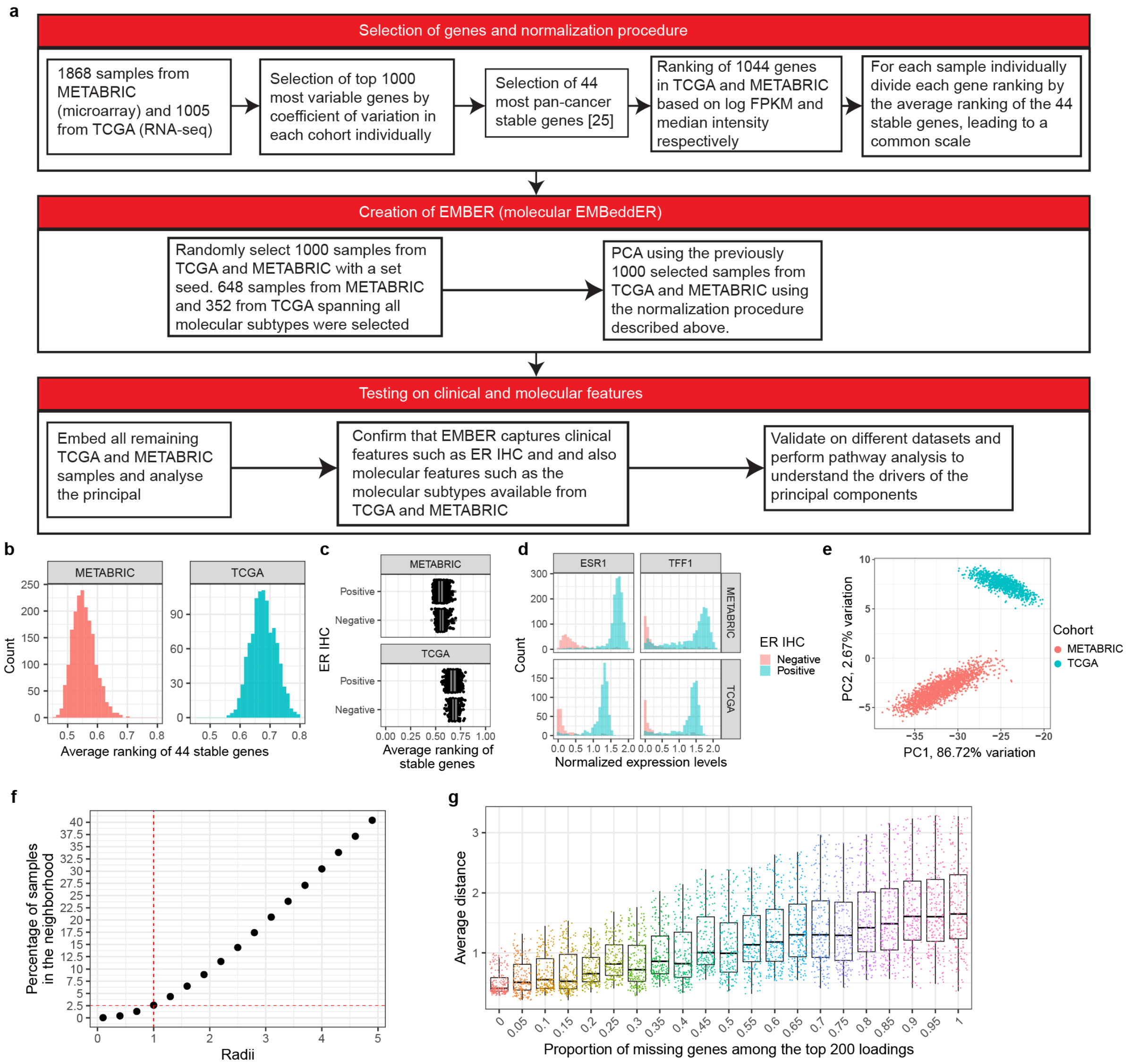

a CONSORT diagram showing how the EMBER was developed. **b** Distribution of the average of the 44 stable genes average rankings (normalized by the total number of genes) stratified by the cohorts for all samples available (TCGA n = 1005; METABRIC n = 1868). **c** Average ranking of stable genes stratified by ER IHC status in METABRIC and TCGA cohorts. The rank of each stable gene was normalized between 0 and 1 dividing by the total number of genes (1044). Each dot represents a single patient sample. **d** Comparison of the normalized ESR1 and TFF1 after the proposed normalization procedure. (TCGA, ER+ tumors n = 774,

ER- n = 231; METABRIC, ER+ tumors n = 1439, ER- n = 231). **e** Score plot of PC1 and PC2 using all samples from METABRIC and TCGA. **f** Average percentage of samples in the neighborhood calculated for 200 random samples from TCGA. **g** Average distance of original embedding to the new embedding when removing 200 genes from the 1044 genes list and with varying proportion of the top 200 loading genes missing. Each dot corresponds to a sample, in total 200 samples from TCGA were used to calculate the distance 10 times each. Average over 10 times for each sample is displayed.

Supplementary Figure 2

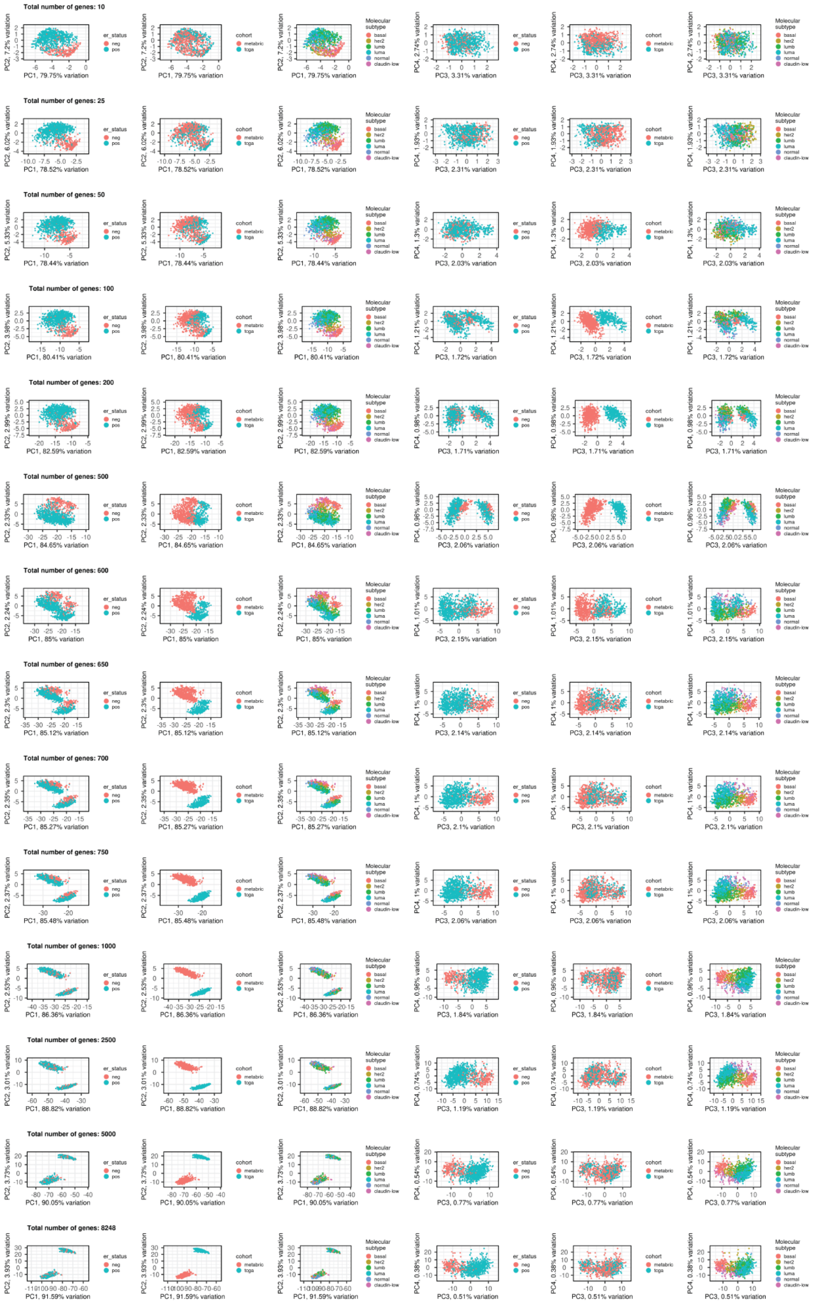

Score plots based on different numbers of genes included. Each row corresponds to a distinct set of genes from 10 up to 8248. From left to right colored by: ER IHC, Cohort, Molecular subtypes of PC1/PC2 and ER IHC, Cohort, Molecular subtypes of PC3/PC4.

Supplementary Figure 3

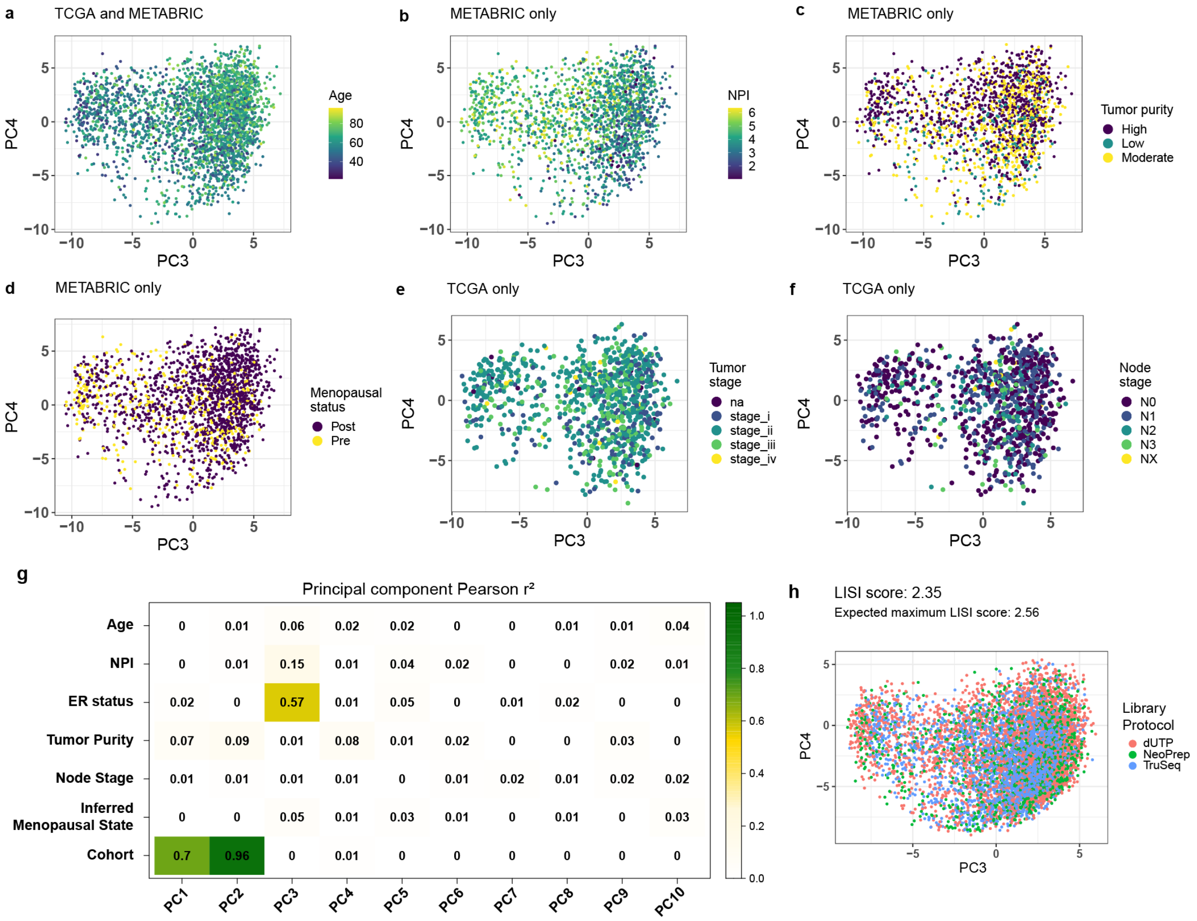

Embedding of all samples from TCGA and METABRIC colored by **a** age. Embedding of all samples from METABRIC only colored by **b** Nottingham Prognostic Index (NPI), **c** tumor purity, **d** and menopausal status. Embedding of all samples from TCGA colored by **e** tumor stage **f** and node stage. **g** R-squared residuals from Pearson correlation among clinical factors/batch effects and the first 10 principal components. **h** Score plot showing EMBER embedding of SCAN-B samples colored by library preparation.

Supplementary Figure 4

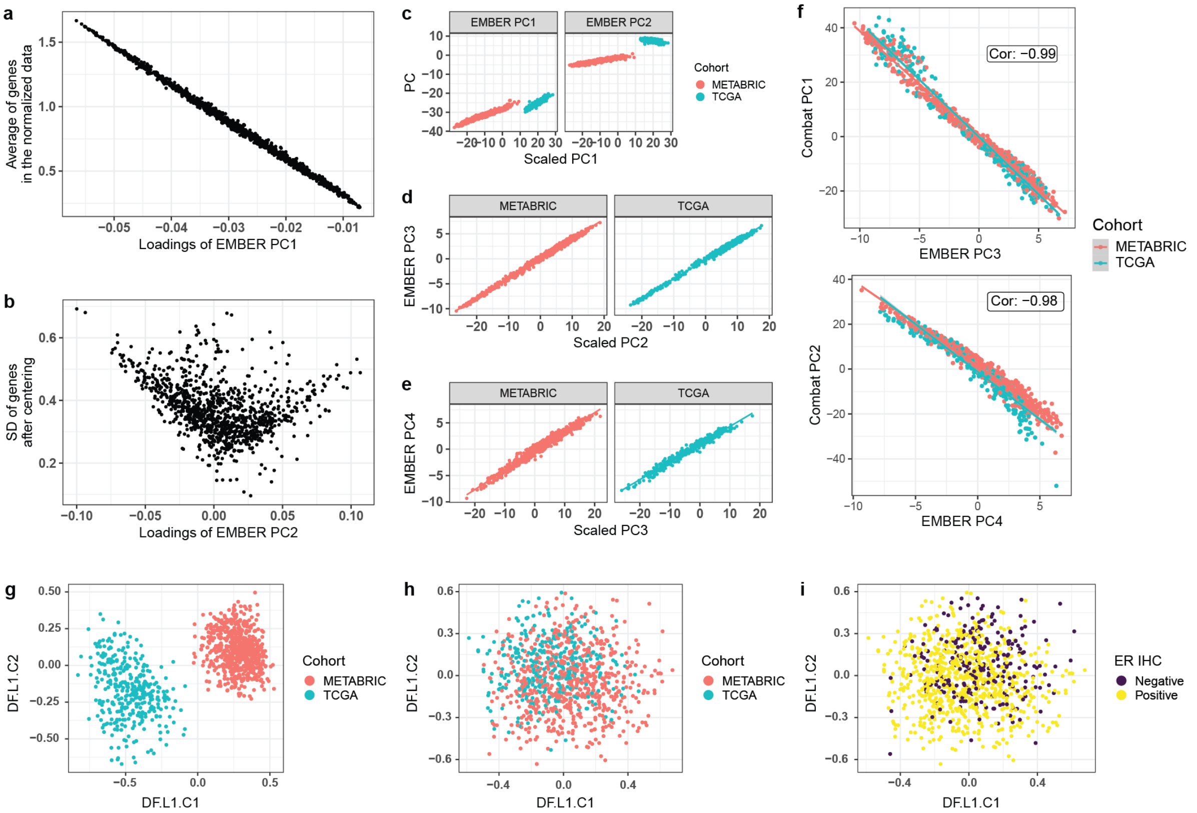

Analysis of centering and scaling the data prior to doing the PCA. **a** Comparison between the loadings of the first component in the original PCA (without any centering and scaling) and the average of the normalized gene expression used for the PCA. **b** same as a but comparing the standard deviation and the loadings of the second component. **c** Comparison between EMBER's PC1 and PC2 with PC1 on the scaled data (setting center and scale to TRUE when using the function PCAtools::pca). **d** Comparison between PC2 from using the scaled data with EMBER PC3 stratified by cohort. **e** Comparison between PC3 from using the scaled data with EMBER PC4 stratified by cohort. **f** Batch removal performance on METABRIC and TCGA samples. Comparison of EMBER PC3/PC4 to Combat's PC1/PC2, respectively. Each dot corresponds to a training sample. Spearman correlation was calculated for each comparison. **g** Encoding of the training samples from TCGA and METABRIC when using an autoencoder with a 2-units hidden layer. **h** Encoding of training samples from TCGA and METABRIC when using a single hidden layer with 4 units colored by Cohort and **i** ER IHC.

Supplementary Figure 5

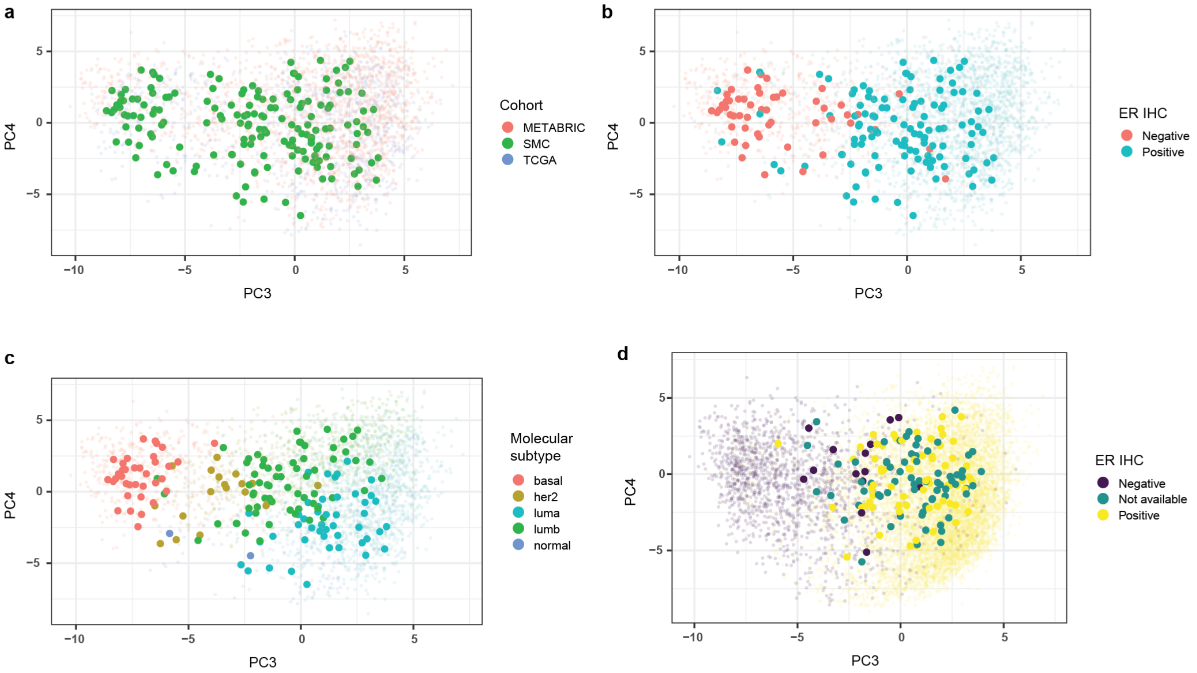

Embedding of all samples from the South Korean BC cohort (SMC) colored by **a** cohort, **b** ER IHC and **c** intrinsic molecular subtype. **d** Embedding of all samples from the Metastatic Breast Cancer Project dataset colored by ER IHC.

Supplementary Figure 6

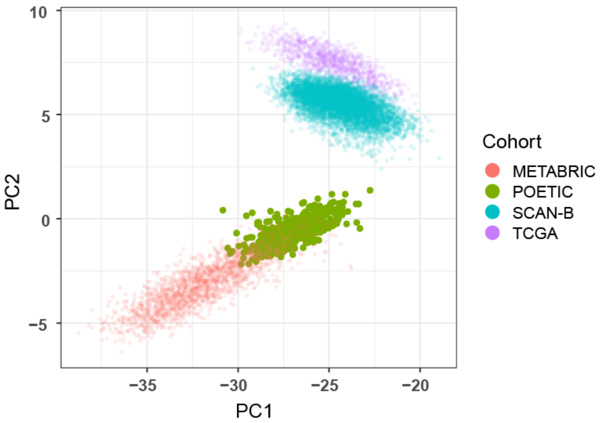

PC1/PC2 EMBER score plot of all samples from TCGA, METABRIC, SCAN-B and POETIC.

Supplementary Figure 7

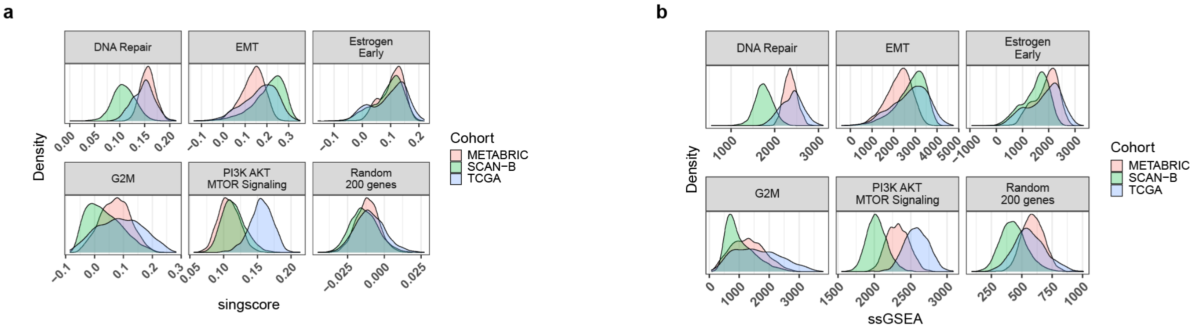

Single sample score distributions for all samples in METABRIC, TCGA and SCAN-B calculated for several molecular pathways using **a** singscore and **b** ssGSEA.

Supplementary Figure 8

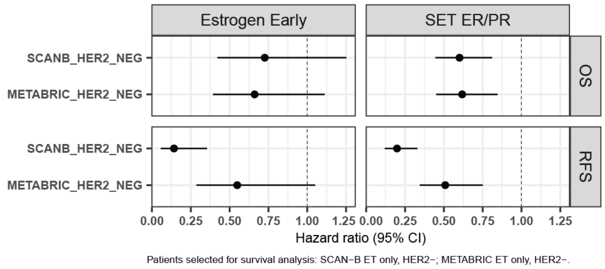

Forest plot of the survival analysis for each cohort separately when including only the ER+ HER2- BC patients that received endocrine therapy only.

Supplementary Table 1

Mean squared errors (MSE) from different Autoencoders architectures.

Architecture	MSE
2	0.049
[100, 2, 100]	0.049
[300, 2, 300]	0.050
[250, 100, 2, 100, 250]	0.050
[50, 2, 50]	0.050
4	0.050
[50, 4, 50]	0.050
[300, 4, 300]	0.050
[100, 4, 100]	0.051
[250, 100, 4, 100, 250]	0.051

Supplementary Table 2

Hazard ratios from the Cox regression survival analysis performed in Fig. 3c.

Type	Cohort	Pathway	Hazard Ratio	p.value	conf.low	conf.high
OS	TCGA	HALLMARK_G2M_CHECKPOINT	2.54	0.03	1.11	5.85
OS	TCGA	HALLMARK_EPITHELIAL_MESENCHYMAL_TRANSITION	1.23	0.55	0.62	2.41
OS	TCGA	HALLMARK_DNA_REPAIR	0.83	0.77	0.23	2.96
OS	TCGA	HALLMARK_ANDROGEN_RESPONSE	1.60	0.47	0.45	5.68
OS	TCGA	HALLMARK_PI3K_AKT_MTOR_SIGNALING	1.95	0.48	0.30	12.77
OS	SCANB	HALLMARK_G2M_CHECKPOINT	1.40	0.08	0.96	2.02
OS	SCANB	HALLMARK_EPITHELIAL_MESENCHYMAL_TRANSITION	1.22	0.11	0.95	1.55
OS	SCANB	HALLMARK_DNA_REPAIR	1.44	0.19	0.83	2.49
OS	SCANB	HALLMARK_ANDROGEN_RESPONSE	1.03	0.91	0.64	1.66
OS	SCANB	HALLMARK_PI3K_AKT_MTOR_SIGNALING	1.85	0.17	0.77	4.45
OS	METABRIC	HALLMARK_G2M_CHECKPOINT	1.68	0.01	1.17	2.42
OS	METABRIC	HALLMARK_EPITHELIAL_MESENCHYMAL_TRANSITION	0.87	0.32	0.66	1.14
OS	METABRIC	HALLMARK_DNA_REPAIR	1.33	0.34	0.74	2.38
OS	METABRIC	HALLMARK_ANDROGEN_RESPONSE	0.82	0.53	0.45	1.51
OS	METABRIC	HALLMARK_PI3K_AKT_MTOR_SIGNALING	2.59	0.04	1.07	6.30
RFS	SCANB	HALLMARK_G2M_CHECKPOINT	5.43	0.00	2.76	10.67
RFS	SCANB	HALLMARK_EPITHELIAL_MESENCHYMAL_TRANSITION	1.37	0.17	0.87	2.14
RFS	SCANB	HALLMARK_DNA_REPAIR	1.44	0.49	0.51	4.05
RFS	SCANB	HALLMARK_ANDROGEN_RESPONSE	3.50	0.00	1.50	8.15
RFS	SCANB	HALLMARK_PI3K_AKT_MTOR_SIGNALING	19.48	0.00	3.92	96.76
RFS	METABRIC	HALLMARK_G2M_CHECKPOINT	2.85	0.00	1.79	4.56
RFS	METABRIC	HALLMARK_EPITHELIAL_MESENCHYMAL_TRANSITION	0.79	0.19	0.55	1.13
RFS	METABRIC	HALLMARK_DNA_REPAIR	2.76	0.01	1.28	5.97
RFS	METABRIC	HALLMARK_ANDROGEN_RESPONSE	0.48	0.07	0.21	1.07
RFS	METABRIC	HALLMARK_PI3K_AKT_MTOR_SIGNALING	4.41	0.01	1.43	13.58
